# Decoupling Activity and Specificity in Coronazymes

**DOI:** 10.1002/smll.202500783

**Published:** 2025-03-04

**Authors:** Jiahao Ji, Li Zuo, Bishal Pokhrel, Pravin Pokhrel, Sajan Shakya, Hao Shen, Hanbin Mao

**Affiliations:** ^1^ Department of Chemistry & Biochemistry Kent State University Kent OH 44242 USA; ^2^ College of Chemistry and Chemical Engineering Xiamen University Xiamen 361005 China; ^3^ Advanced Materials and Liquid Crystals Institute Kent State University Kent OH 44242 USA; ^4^ School of Biomedical Sciences Kent State University Kent OH 44242 USA

**Keywords:** activity and specificity, chiral modulation, CISS, modular design, mosaicking

## Abstract

Specificity and activity are often at odds for natural enzymes. In this work, specificity and activity in coronazymes made of an Au nanoparticle (AuNP) and coated with DNA aptamer for glucose substrates are decoupled. By single‐molecule fluorescent MT‐HILO (magnetic tweezers coupled with highly inclined and laminated optical sheet) microscopy, it is found that this coronazyme has ≈30 times higher activity on the d‐glucose compared to bare AuNP nanozymes. Significantly, the new coronazyme demonstrates long‐range modulations by circularly polarized light (CPL) according to the matching chirality between the CPL and DNA corona, which follows the rule of chiral induced spin selectivity (CISS). Although the aptamer in the coronazyme is evolved against d‐glucose, surprisingly, this coronazyme catalyzes l‐glucose better than d‐glucose, likely due to the faster rates for the aptamer to interact with the l‐ over d‐glucose. These results demonstrate, for the first time, an artificial enzyme with its catalytic activity controlled by short‐range intermolecular forces, whereas its chiral specificity is modulated by long‐range CPLs. This decoupled arrangement is pivotal to forge premier catalysts with activity and specificity superior to natural enzymes by separately optimizing these two properties.

## Graphical abstract

A new Au nanoparticle (AuNP) based nanozyme, aptamer–coronazyme, which demonstrates ≈30 times higher activity on d‐glucose than the pristine AuNP nanozymes, is designed and characterized. This coronazyme shows long‐range chiral specificity controlled by circularly polarized lights and catalytic reactivity modulated by short‐range intermolecular forces. This activity and specificity decoupling in coronazymes is pivotal to forge premier catalysts in future.

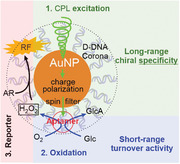

## 1 Introduction

Interactions between enzymes and substrates are governed by short‐range intermolecular forces (IMF).^[^ ^1^ ^]^ High IMF results in tight binding of substrates to enzymes, which causes high specificity in enzymes. This high specificity, however, may not lead to high turnover rates: a tight binding often comes with a slow off‐rate that decreases enzyme activity. Therefore, in natural enzymes, activities and specificity are coupled as both are dependent on the IMF.

This coupling makes it rare for an enzyme to have both high specificity and high activity. For example, nonspecific enzymes such as peroxidase^[^ ^2^ ^]^ and phosphatase^[^ ^3^ ^]^ have rather fast catalytic activities, whereas enzymes/ribozymes that cleave nucleic acids at specific sequences are slow in turn‐over rates.^[^ ^4^ ^]^ This trend persists within the same class of enzymes: lyases that work on different molecular substrates^[^ ^5^ ^]^ exhibit faster turnover rates compared to those with more specific substrates, such as DNA.^[^ ^6^ ^]^ In this work, we attempt to decouple these two properties by modulating specificity with long‐range chiral factors, while keeping the catalytic activities controlled by short‐range IMF.

One example of long‐range chiral interactions is the electron/charge transfer that takes place during chiral light–matter communications. It has been demonstrated that chiral induced spin selectivity (CISS)^[^ ^7^ ^]^ is able to polarize certain spin states of electrons according to the chirality of hosting molecules.^[^ ^8^ ^]^ These electrons then participate in subsequent redox reactions with different activities. However, there are only scattered reports^[^ ^8^ ^]^ using long‐range chiral interactions to control activities of redox catalysts.^[^ ^9^ ^]^ While many natural enzymes are chiral, they have rather complicated structural and functional properties that obscure the potential long‐range chiral modulation effect. For instance, in redox enzymes, the active site often constitutes only a fraction of the whole structure, making it rather challenging to vary the route of electron transfer without completely altering structures or functions of enzymes. In contrast, artificial enzymes such as DNAzymes,^[^ ^10^ ^]^ nanozymes,^[^ ^11^ ^]^ and coronazymes^[^ ^12^ ^]^ have simpler structures, causing easier variation in their structures to test chiral modulation principles. In addition, artificial elements such as nanoparticles in these synthetic enzymes can be readily exploited to respond to circularly polarized lights for subsequent chiral modulations.

**Figure 1:**
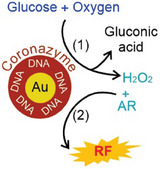
A cascade reaction catalyzed by a glucose aptamer–coronazyme. Coronazyme catalyzes oxidation of glucose to produce H_2_O_2_ (step [1]), which then oxidizes AR (amplex red) to RF (resorufin), a fluorescence product excited by the 532 nm light (step [2]).

Here, based on a framework of coronazyme, an artificial enzyme in which an Au nanoparticle (AuNP) core (or a nanozyme) is coated with a DNA corona (**Figure** **1**),^[^ ^12^ ^]^ we mosaicked three different chiral elements, namely circularly polarized light (CPL), DNA aptamer based corona, and an enantiomer substrate recognized by the aptamer, into a new class of coronazyme. The coronazyme exhibited glucose oxidase mimicking capability and outperformed bare nanoparticles in reactivity and selectivity.^[^ ^13^ ^]^ During the catalytic transformation, a two‐step cascade reaction occurred, starting with glucose oxidation, followed by conversion of Amplex Red (AR) into resorufin (RF), a fluorescent product (Figure 1). By evaluating catalytic activities, we revealed the mosaicked coronazyme had drastically improved its catalytic activity (≈30 times) on the cascade reaction compared to the pristine AuNP nanozyme. More importantly, we confirmed that long‐range CPLs indeed modulate chiral specificity of the coronazyme, whereas short‐range intermolecular force controls turnover activities of the coronazyme. The decoupled activities and specificities, therefore, allow us to separately perfect these two properties in the coronazyme, a feature surpassing natural enzymes.

## 2 Results and Discussion

### 2.1 A Mosaic Framework of Aptamer–Coronazymes

The gold nanoparticle (AuNP) core in a coronazyme has demonstrated glucose oxidase activity.^[^ ^14^ ^]^ We therefore used the coronazyme to catalyze the oxidation of glucose to produce hydrogen peroxide, which then turned over a nonfluorescent molecule amplex red (AR) to resorufin (RF), a fluorescent molecule (Figure 1) whose 583 nm emission can be measured in a single‐molecule fluorescent MT‐HILO microscope (see Sections S1 and S4, Supporting Information for details).^[^ ^15^ ^]^ As AuNP can catalyze the turnover of the AR to RF rapidly,^[^ ^12^ ^]^ the fluorogenic reaction can be conveniently used to report the oxidation reaction of the glucose catalyzed by the coronazyme.

**Figure 2:**
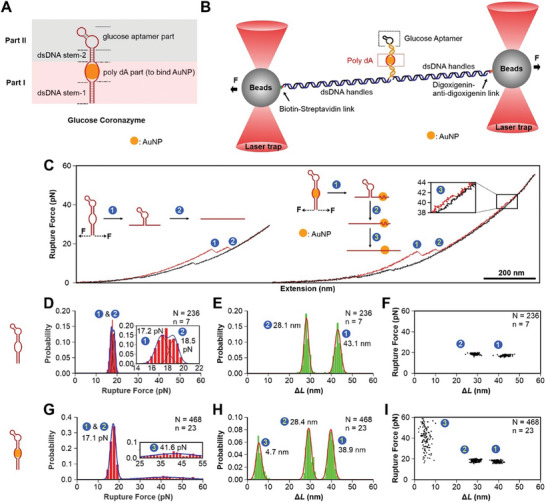
Structures of single aptamer–coronazymes revealed by mechanical unfolding experiments. A) Schematic of aptamer–coronazyme and its components. B) Setup of mechanical unfolding of aptamer–coronazyme using optical tweezers. C) Typical F–X curves of aptamer–coronazyme without (left) and with AuNP (right) marked with unfolding steps. Rupture force (D&G) and Δ*L* (E&H) histograms, and the corresponding scatter plots (F&I) of aptamer–coronazymes without and with AuNP cores. The experiments were performed at 23 °C in 20 mm HEPES buffer complemented with 10 mm MgCl_2_, 5 mm KCl, and 1 m NaCl at pH 7.5.

To specifically recognize glucose substrate, we introduced an aptamer^[^ ^16^ ^]^ in the DNA corona of coronazyme as a specific d‐glucose binding site^[^ ^17^ ^]^ (See **Figure** **2**A; Figures S1 and S5, Supporting Information). To facilitate the catalysis of the glucose by the coronazyme, we added the glucose aptamer between the two poly(dA)_21_ segments that bound to the AuNP core in the coronazyme (Figure 2A).

Next, we carried out mechanical unfolding in optical tweezers to characterize the structures of as‐prepared aptamer–coronazyme (Figure 2B; see Sections S1 and S7, Supporting Information for details). To this end, the coronazyme construct was tethered between two optically trapped polystyrene beads via two dsDNA handles. When we moved one of the optically trapped beads away from the other, the coronazyme experienced increased tension. Once the tension was higher than the mechanical stability of the DNA structure in the coronazyme coating, rupture events occurred in force versus extension (F–X) curves, from which we measured the rupture force (which is related to the mechanical stability) and corresponding change‐in‐contour‐length (Δ*L*, which is related to the structure size) for each rupture event according to reported procedures.^[^ ^18^ ^]^

The structure and mechanical properties of the coronazyme without aptamer (i.e., wild‐type [WT] coronazyme) have been studied in previous work.^[^ ^12^, ^19^ ^]^ For the coronazyme with glucose aptamer (aptamer–coronazyme), the mechanical unfolding experiments revealed two features with rupture forces (pN) and changes‐in‐contour‐length (Δ*L*) located at 17.2 pN/43.1 nm and 18.5 pN/28.1 nm (Figure 2C–F). Due to the geometry of the mechanical pulling, Part I of the DNA construct (Figure 2A) bore the full tension; and therefore, unfolded first (17.2 pN/43.1 nm) before the Part II (18.5 pN/28.1 nm). Δ*L* values of the Part I/II segments are close to expected Δ*L*s (for calculations, see Section S8, Supporting Information), showing the success of the coronazyme preparation.

After confirming the structure of the aptamer–coronazyme without AuNP, we investigated the aptamer–coronazyme in the presence of AuNP using the same mechanical unfolding approaches. With freeze–thaw method,^[^ ^20^ ^]^ two poly(dA)_21_ segments in the corona DNA bound to a 5‐nm AuNP.^[^ ^12^, ^19^ ^]^ With the increasing force exerted on the aptamer–coronazyme with AuNP (Figure 2C), Part I of the coronazyme was unfolded first (step 1), followed by the unfolding of the DNA stem in Part II (step 2), and the subsequent stretching of the poly(dA)_21_ loop attached on the AuNP surface in Part II (step 3). These three steps showed rupture forces and corresponding Δ*L* values of 17.1 pN/38.9 nm (step 1), 17.1 pN/28.4 nm (step 2), and 41.6 pN/4.7 nm (step 3), respectively (Figure 2G–I). Our measured unzipping force aligned well with previous studies, demonstrating the mechanical stability of the coronazyme.^[^ ^21^ ^]^ As observed Δ*L* values matched with expected Δ*L* calculations (see Section S8, Supporting Information for details), it confirmed the expected structure of the aptamer–coronazyme. In particular, the 41.6 pN/4.7 nm feature was consistent with that observed in the WT‐coronazyme without aptamer,^[^ ^19^ ^]^ which has been ascribed to the stretching of the poly(dA)_21_ section on AuNP surface, characteristic of the presence of AuNP in the coronazyme.

### 2.2 Catalysis of a Cascade Reaction by Individual Aptamer–Coronazymes

**Figure 3:**
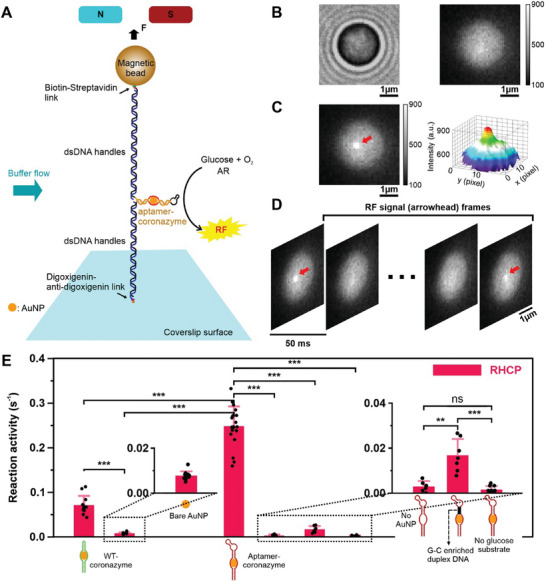
Single‐molecule fluorescence experiments to measure catalytic activities of individual aptamer–coronazymes. A) Setup of single‐molecule fluorescence MT‐HILO experiment. The aptamer–coronazyme was fixed on the coverslip surface and tethered to a magnetic bead via two dsDNA handles. The orientation of the coronazyme was maintained by a constant flow of reaction mixture at 5 µL min^−1^. B) The bright‐field (left) and the fluorescence (right) images of an aptamer–coronazyme linked magnetic bead. C) Typical fluorescence signal (marked by an arrowhead) of the RF product catalyzed by the aptamer–coronazyme (left) and its 3D histogram (right). The size of each pixel was equal to 108 nm × 108 nm. D) Temporal image frames with single‐molecular RF signals catalyzed by the aptamer–coronazyme. The exposure time for each image frame was 50 ms. E) The catalytic activities of different coronazymes under right‐handed circularly polarized light (RHCP). From left to right: WT‐coronazyme, 5 nm bare AuNP, aptamer–coronazyme, aptamer–coronazyme without AuNP, aptamer–coronazyme with a G–C enriched duplex DNA stem and an internal loop (for black section, see Figure S7, Supporting Information for details), and aptamer–coronazyme without glucose substrate. Error bars depict the standard deviations from at least ten catalysts that were collected for each sample group. The reactions were performed at 23 °C with 10 mm d‐glucose in 20 mm HEPES buffer complemented with 10 mm MgCl_2_, 5 mm KCl, and 1 m NaCl at pH 7.5. These coronazymes were exposed to 3 mW RHCP/LHCP during the experiments. The one‐tail unpaired *t*‐test was used to compare data points. The ns means *p* > 0.05, ** means *p* < 0.01, and *** means *p* < 0.001.

Next, we evaluated catalytic activities of individual aptamer–coronazymes in a single‐molecule fluorescence MT‐HILO microscope.^[^ ^15a^ ^]^ To this end, the aptamer–coronazyme was ligated with two dsDNA handles, one of which was attached to the inner surface of a microfluidic chamber via digoxigenin–anti‐digoxigenin linkage, while the other was bound to a magnetic bead through the biotin–streptavidin linkage (**Figure** **3**A,B). Using magnetic tweezers, we maintained a minimal tensile force of 1.0 pN for the entire DNA construct; so that, the AuNP in the middle of the coronazyme was isolated from the chamber surface where background fluorescence signal was ample. Such a levitation also increased the mass transfer of substrates and products, facilitating the catalytic turnovers.^[^ ^15a^ ^]^ To start the catalytic reaction, we flowed in 10 mm d‐glucose and 0.5 µm AR in HEPES buffer (20 mm HEPES buffer complemented with 10 mm MgCl_2_, 5 mm KCl, and 1 m NaCl at pH 7.5) with 50% saturated O_2_ gas (≈0.1 mm ^[^ ^22^ ^]^). After d‐glucose was oxidized by the coronazyme, H_2_O_2_ was released. This led to the coronazyme catalyzed conversion of the AR to the RF product (Figures 1 and 3C). The single‐molecule RF fluorescence signals were recorded over time in the fluorescence microscope (Figure 3D), which was used to calculate the reaction rate constants. Given that AuNP itself can catalyze AR to RF, a control experiment (aptamer–coronazyme with AR but without d‐glucose) was conducted to ensure that the observed fluorescent signals originated from glucose oxidation rather than from direct catalysis by AuNP. The extremely low catalytic activity (0.002 ± 0.0005 s⁻¹, Figure 3E, right) confirmed the source of the fluorescent signals.

As shown in Figure 3E, the WT‐coronazyme has a catalytic activity of 0.071 ± 0.021 s^−1^, around nine times higher than bare AuNP (0.008 ± 0.002 s^−1^) for this cascade reaction on d‐glucose substrate under RHCP (Figure 1). On the other hand, the aptamer–coronazyme showed the highest activity of 0.248 ± 0.044 s^−1^ (Figure 3E) with high enzymatic stability (Figure S8, Supporting Information), which was ≈30 times higher than the activity of pristine AuNP. The aptamer and AuNP synergistically increased the reactivity by ≈four times compared to WT‐coronazyme. This unprecedented activity improvement may be due to three reasons. First, the glucose aptamer itself can catalyze glucose turnover. Second, the aptamer binds to glucose molecules, increasing the local concentration that enhances apparent catalytic activity. Third, aptamer–coronazyme can turnover glucose more efficiently due to the charge transfer mechanism as discussed in previous work.^[^ ^12^ ^]^

To identify the role of glucose aptamer in the coronazyme, we first ruled out the catalytic capability of the DNA corona (reason 1), which showed a value (0.003 ± 0.002 s^−1^) close to the background (Figure 3E). To differentiate the remaining two reasons, we changed the DNA sequence between the glucose aptamer and the AuNP binding poly(A)_21_ segments (shown in Figure S7, Supporting Information) to G–C enriched sequence, which was expected to inhibit the charge transfer in duplex DNA.^[^ ^23^ ^]^ In addition, an internal loop (Figure S7, Supporting Information) was introduced in the same section to further disrupt the charge transfer.^[^ ^24^ ^]^ When we measured the catalytic activity for this coronazyme, we found ≈15 times activity decrease from 0.248 to 0.017 s^−1^, which supported the involvement of the charge transfer mechanism (reason 3) in the catalysis. The fact that aptamer–coronazyme (0.248 ± 0.044 s^−1^) has four times higher activity than WT‐coronazyme (0.071 ± 0.021 s^−1^) suggests that glucose aptamer is an important factor in the catalysis, likely due to its glucose enrichment effect (reason 2).

### 2.3 Long‐Range Modulation of Chiral Specificities Via the CISS Effect

**Figure 4:**
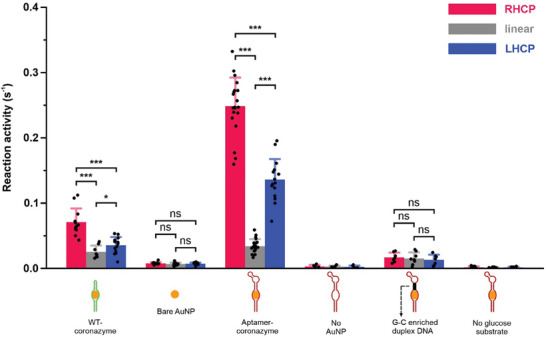
CISS effect on the chiral specificity of coronazyme catalysis. From left to right: WT‐coronazyme, 5 nm bare AuNP, aptamer–coronazyme, aptamer–coronazyme without AuNP, aptamer–coronazyme with a G–C enriched DNA section, and aptamer–coronazyme without glucose. Error bars depict the standard deviations from at least ten catalysts for each sample group. The reactions were performed with 10 mm d‐glucose in 20 mm HEPES buffer complemented with 10 mm MgCl_2_, 5 mm KCl, and 1 m NaCl at pH 7.5. These coronazymes were exposed to 3 mW RHCP/LHCP during the experiments. (LHCP: left‐handed circularly polarized light) The one‐tail unpaired *t*‐test was used to compare data points. The ns means *p* > 0.05, * means *p* < 0.05, and *** means *p* < 0.001.

Chirality induced spin selectivity (CISS) describes the coupling between electron spin and chiral framework of a molecule.^[^ ^7b^ ^]^ Although AuNP is achiral per se, when a chiral material such as DNA is attached to the AuNP surface, spin–orbit coupling (SOC) and orbital polarization effect (OPE) would encode chirality to the AuNP due to the chiral corona DNA.^[^ ^8^, ^25^ ^]^ This chiral coronazyme may therefore respond differently to the circularly polarized light (CPL) with different handedness (i.e., chirality). The hot electrons produced at the AuNP–DNA interface would exhibit a spin preference which facilitates the transfer of these electrons to the DNA strand, while the electrons with mismatched spin state are bounced back until they flip their spin status.^[^ ^26^ ^]^ Such a behavior (matching in the electron spin state and DNA chirality) allows us to evaluate the chiral matching principle via the CISS effect (**Figure** **4**).

For WT‐coronazymes (Figure 4), the reaction activities are 0.071 ± 0.021 s^−1^, 0.035 ± 0.013 s^−1^, and 0.025 ± 0.010 s^−1^ under the RHCP, LHCP, and linear light, respectively. Similar trends (RHCP [0.248 ± 0.044 s^−1^] > LHCP [0.136 ± 0.032 s^−1^] > linear light [0.034 ± 0.011 s^−1^]) were observed in aptamer–coronazymes. In contrast, 5 nm bare AuNP (≈0.01 s^−1^) and G–C enriched aptamer–coronazyme responded similarly to RHCP (0.017 ± 0.007 s^−1^), LHCP (0.013 ± 0.008 s^−1^), and linear light (0.015 ± 0.009 s^−1^). The general trend observed for various coronazymes was likely due to the CISS effect.^[^ ^8^, ^27^ ^]^

**Figure 5:**
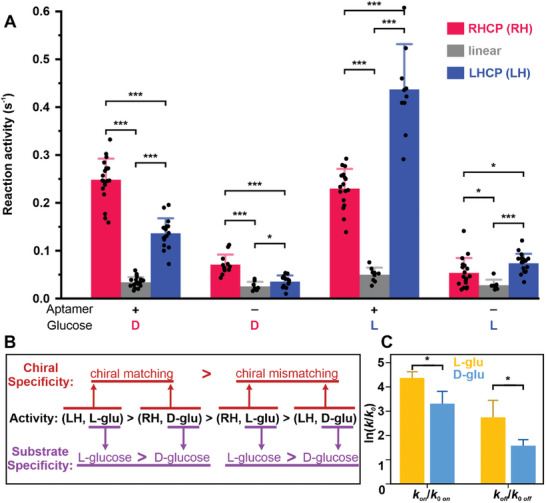
Substrate effect on the turnover activity of coronazyme catalysis. A) Activities of different coronazymes with 10 mm d‐/l‐glucose. Error bars depict the standard deviations from at least 14 catalysts for each condition. B) Schematic of the short‐range substrate effect and the long‐range chirality effect on the coronazyme activity. RHCP and LHCP are designated as RH and LH, respectively, whereas d‐ and l‐ glucoses are designated as d‐glu and l‐glu respectively. Chiral matching refers to either (LH, l‐glu) or (RH, d‐glu) whereas chiral mismatching refers to either (RH, l‐glu) or (LH, d‐glu). C) Folding (*k*_on_) and unfolding (*k*_off_) rate constants of 2 µm glucose aptamer in the presence of 100 mm d‐ or l‐glucose at 23 °C. Standard deviations (error bars) were obtained from three independent UV melting measurements. The one‐tail unpaired *t*‐test was used to compare data points. The * means *p* < 0.05 and *** means *p* < 0.001. The experiments were carried out at 23 °C in 20 mm HEPES buffer supplemented with 10 mm MgCl_2_, 5 mm KCl, and 1 m NaCl at pH 7.5. The *k*_0_ depicts the folding or unfolding rate constant of 2 µm glucose aptamer without glucose in the HEPES buffer.

Chiral DNA not only induces electron spin polarization at the interface but also acts as a spin filter, allowing only electrons with matching spin directions to pass.^[^ ^7^, ^8^ ^]^ Consequently, the amount of electrons reaching to the DNA‐bound substrate would be different if the electron spin preference at the interface is modulated through different CPL excitations.^[^ ^8^ ^]^ For WT‐ or aptamer‐coronazymes with d‐glucose, such a CISS mediated filtering effect leads to polarizations of +33.3% and +29.2%, respectively. It is noteworthy that we have used reactivity polarization to reflect the spin polarization during the catalysis. The polarization was calculated by *p*  =  (ν_RHCP_ − ν_LHCP_)/(ν_RHCP_ + ν_LHCP_), where *v* is the reaction rate constant under specific conditions (for calculation details, see Section S11, Supporting Information). The positive polarizations under RHCP are likely because RHCP polarized electron spin is aligned better with the chirality of the d‐DNA and d‐glucose than the LHCP. If such a chirality matching principle does exist, then negative polarizations are expected when l‐glucose is used as a substrate. Indeed, these were observed in **Figure** **5**A, where polarizations were −15.8% for WT coronazyme and −31.1% for aptamer–coronazyme, confirming the chiral specificity of this coronazyme. The spin polarizations of aptamer–coronazymes (+29.2% with 10 mm d‐glucose and −31.1% with 10 mm l‐glucose) were significantly higher than those of natural glucose oxidase (≈5–10% with 10 mm d‐glucose and ≈0–3% with 10 mm l‐glucose),^[^ ^9^ ^]^ indicating a superior capability of CISS‐mediated filtering effect in aptamer–coronazymes.

To investigate the factors controlling the chiral specificity, we have compared the coronazyme activities under different CPLs (RHCP and LHCP) and substrate chiralities (d‐ and l‐ glucose) (Figure 5A,B). We observed that the matched chiralities between CPL and glucose, that is, (RHCP + d‐glucose) or (LHCP with l‐glucose), presented higher coronazyme activities than mismatched chiralities, that is, (RHCP + l‐glucose) or (LHCP with d‐glucose). Such a trend can be explained by the CISS effect in which transportation of specifically polarized electron spins is facilitated between different components with matching chiralities (see above). Compared to the chiral specificity in natural enzymes, which is often achieved via short‐range intermolecular interactions (i.e., IMF) between biomolecules and substrates, the strong chiral specificity achieved by CPL demonstrated here offers unprecedented opportunity to modulate chiral specificity of catalysts in a long‐range manner.

It is surprising that under all conditions, coronazymes have the lowest activity under linear light. As linear light is a mixture of RHCP and LHCP, the activity under the linear light is expected to be located between RHCP and LHCP. To explain this observation, we noticed there is a cross‐over of electron spins during this reaction (Figure S9, Supporting Information). During catalysis, oxygen molecules were converted to H_2_O_2_. As a result, the unpaired electron spins in oxygen became paired in H_2_O_2_, which is an energetically unfavorable spin‐forbidden process.^[^ ^28^ ^]^ As CPLs polarize electron spins, the spin forbidden process can be avoided, facilitating the coronazyme catalyzed reactions. In contrast, linear light cannot polarize electron spins and the spin forbidden process persists, reducing the catalytic rate of the coronazyme. A similar phenomenon has been observed in literature.^[^ ^29^ ^]^

### 2.4 Kinetic Interaction Between Chiral Substrate and Aptamer–Coronazyme Determines Catalytic Activities

It is surprising that coronazymes showed higher catalytic activities for the l‐glucose substrate compared to the d‐glucose, regardless of different light sources (CPLs or linear light) or modification states of coronazymes (Figure 5A,B). Given that the aptamer was obtained by screening against d‐glucose,^[^ ^17^ ^]^ the binding affinity of the aptamer should be stronger for the d‐glucose than the l‐glucose. Thus, the higher catalytic activities observed for the l‐glucose were likely due to the kinetic preference of the aptamer toward the l‐glucose rather than the d‐glucose (i.e., binding or dissociation of the l‐glucose to or from the aptamer is faster than the d‐glucose). To test this hypothesis, we performed UV melting of the aptamer and calculated the folding and unfolding rate constants of the aptamer with and without d‐ or l‐glucose (Figure 5C; see Sections S1 and S5, Supporting Information for details). By comparing these folding or unfolding rate constants with and without ligands, we were able to evaluate the effect of the ligand on the folding or unfolding process of the aptamer. We found that l‐glucose induced faster folding and unfolding of the aptamer than d‐glucose, which likely reflected the higher interaction probability between the l‐glucose and the aptamer with respect to the d‐glucose.

**Figure 6:**
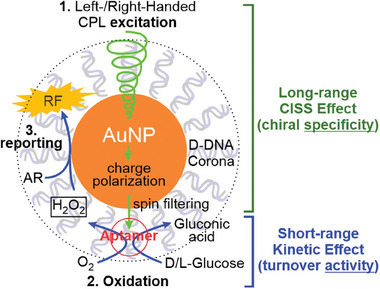
Chiral specificity and turnover activity are decoupled in aptamer–coronazyme catalysis.

Taking together, we propose a three‐stage mechanism to explain the modulations of all three chiral elements during the aptamer–coronazyme catalysis (**Figure** **6**). In the first stage, the first chiral element, CPL, induces charge polarization on the AuNP core via a photoelectron effect.^[^ ^30^ ^]^ The polarized charge is then propagated and filtered by the second chiral element, chiral DNA molecules, at the coronazyme surface due to the CISS effect.^[^ ^8^ ^]^ In the second stage, the third chiral element, glucose, binds to the aptamer in the corona layer. The different kinetic effects on the binding and dissociation of the chiral glucose molecules to the aptamer explain the differential activities of the aptamer–coronazyme. In the third stage, the polarized charge reaches glucose‐bound aptamers, overcoming the electron spin forbidden barrier during the conversion of O_2_ to H_2_O_2_, which takes place in the vicinity of AuNP surface. Finally, the H_2_O_2_ helps to turnover the fluorogenic reaction of AR to RF, which is also catalyzed by AuNP in the aptamer–coronazyme. Compared to other artificial enzymes, the coronazyme presented here offers a unique design where the AuNP serves as the charge center, continuously providing electrons and holes for substrate conversion. Simultaneously, an integrated aptamer in the corona phase acts as a specific substrate binding site, enhancing reaction selectivity. The separation of the charge center from the reaction site enables precise control over both chiral specificity and catalytic activity by CISS mediated long‐range charge transfer and IMF mediated short‐range intermolecular interactions, respectively. Such a framework distinguishes coronazymes from natural enzymes whose specificity and activity are often at odds.

## 3 Conclusions

In summary, after mosaicking three chiral elements of CPL, coronazyme, and substrate in a coronazyme framework, we demonstrated a new artificial enzyme with its catalytical activity drastically higher (≈30 times) than the pristine AuNP nanozyme core. More importantly, we confirmed that the chiral specificity of this mosaicked coronazyme was modulated by long‐range CPLs via the CISS effect, whereas its catalytic activity was separately controlled by short‐range IMF interactions between the substrate and DNA aptamer in the corona coating. The decoupled chiral specificity and catalytic activity provided convincing evidence that mosaicking different elements is effective in forging artificial enzymes into a machinery superior to natural enzymes where specificity and activity are often mutually exclusive. We anticipate that by incorporating chiral components for improved charge transfer, utilizing aptamers as specific substrate recognition, and employing external factors such as light and magnetic fields for long‐range modulations, artificial enzymes will exhibit hitherto unseen catalytic performance with respect to natural enzymes.

## 4 Experimental Section

### Chemicals and Reagents

All chemicals and reagents, if not specifically mentioned, were purchased from Thermo Fisher (www.thermofisher.com), Sigma–Aldrich (www.sigmaaldrich.com), or VWR (www.vwr.com). Enzymes were procured from New England Biolabs (NEB). DNA Oligos were obtained from Integrated DNA Technologies (www.idtdna.com) and purified by PAGE (for more details, see Section S1, Supporting Information).

### Synthesis of Coronazymes

The coronazyme was made of the 5 nm bare gold nanoparticle (AuNP) and corona DNA through Au–Poly(dA) interactions. The 5 nm bare AuNPs were obtained from nanoComposix (San Diego, CA, USA). To prepare the corona DNA of coronazymes, DNA oligos (Oligos I–V; for sequences, see Table S1, Supporting Information) were phosphorylated, followed by gradual annealing from 95 °C to 20 °C at a rate of 1 °C min^−1^. The hybridized DNA was ligated via T4 DNA ligase at 16 °C for 16 h (Figure S1, Supporting Information). The DNA corona sample was subsequently annealed with two primers (for sequences, see Table S1, Supporting Information) and finally ligated with 2391 bp dsDNA handles and 1558 bp dsDNA handles (for more details, see Sections S1 and S2 and Figure S1, Supporting Information). To synthesize coronazymes, DNA corona samples and 5 nm bare AuNPs were mixed at 10:1 mole ratio, placed in −80 °C for 30 min, and finally, thawed at room temperature (Figure S2, Supporting Information). The final mixture solution containing coronayzmes was stored at 4 °C.

### Experimental Procedures

Single‐molecule mechanical unfolding experiment setup in optical tweezers is described in Sections S1 and S3, Supporting Information. The single‐molecule fluorescent experiments setup is shown in Sections S1 and S4, Supporting Information. UV melting experiments and related calculations are reported in Sections S1 and S5, Supporting Information.

### Analytical Methods

The raw data about single‐molecule mechanical unfolding was recorded in a Labview 8 program and treated by Matlab and Igor (WaveMetrics) software. The raw data of single‐molecule fluorescent experiments was saved through the Olympus CellSens Dimension software, and then, analyzed using ThunderSTORM. The expected Δ*L* calculations were described in Section S8, Supporting Information.

## Conflict of Interest

The authors declare no conflict of interest.

## Supporting information



Supporting Information

## Data Availability

The data that support the findings of this study are available in the Supporting Information of this article.
