# Supplementary material for: Decoupling Activity and Specificity in Coronazymes
Source: Small. 2025 Mar 4;21(14):2500783. doi: 10.1002/smll.202500783 (PMC11983253; doi:10.1002/smll.202500783)
Supplement: Supplementary file 1 — Supporting Information [file SMLL-21-2500783-s001.pdf]

NANO · MICRO
small

Supporting Information

for *Small*, DOI 10.1002/smll.202500783

Decoupling Activity and Specificity in Coronazymes

Jiahao Ji, Li Zuo, Bishal Pokhrel, Pravin Pokhrel, Sajan Shakya, Hao Shen and Hanbin Mao**

Supporting information

Decoupling Activity and Specificity in Coronazymes

Jiahao Ji,[1] Li Zuo,[1,2] Bishal Pokhrel,[1] Pravin Pokhrel,[1] Sajan Shakya,[1] Hao Shen,[1,*]

and Hanbin Mao[1,3,4,*]

[1]Department of Chemistry & Biochemistry, Kent State University, Kent, OH 44242, USA

[2]College of Chemistry and Chemical Engineering, Xiamen University, Xiamen, 361005, China

[3]Advanced Materials and Liquid Crystals Institute, Kent State University, OH 44242, USA

[4]School of Biomedical Sciences, Kent State University, OH 44242, USA

[*] Corresponding author: Hao Shen (hshen7@kent.edu) and Hanbin Mao (hmao@kent.edu)

Table of contents

Contents

S1. Methods and Materials 3

S2. Syntheses of DNA samples and coronazymes 7

S3. Microfluidic chamber for optical tweezers experiments 9

S4. Single-molecule fluorescent MT-HILO microscopy 11

S5. The folding (k_{on}) and unfolding (k_{off}) rate constants calculations 12

S6. DNA corona structure of aptamer-coronazyme 13

S7. Mechanical unfolding results correlated with glucose binding 14

S8. Expected ΔL calculations 14

S9. DNA corona structure of G-C enriched aptamer-coronazyme 16

S10. Enzymatic stability test 16

S11. Spin polarization calculation 16

S12. Electron spin change during glucose catalysis 17

S13. References 20

S1. Methods and Materials

Methods

Synthesis of DNA constructs

Based on our previous study, a DNA hairpin containing two poly(dA)$_{21}$ internal loops offers the best AuNP binding capability,[1] likely due to cooperative binding effects. To prepare corona DNA used in the coronazyme, first, DNA oligos (Oligos I-V, sequences see Table S1) were phosphorylated, followed by gradual annealing from 95 to 20 °C at a rate of 1 °C/minute. The hybridized DNA was ligated via T4 DNA ligase at 16 °C for 16 hours (NEB, Figure S1). The DNA corona sample was subsequently annealed with two primers (sequences see Table S1) by the same temperature ramping procedure and ligated with the 1558 bp dsDNA handle, whose end was modified with biotin, and the 2391 bp dsDNA handle, whose end was modified with digoxigenin, in a one-pot reaction catalyzed by the T4 DNA ligase at 16 °C for 16 hours (Figure S1). The 1558 bp & 2391 bp dsDNA handles were prepared by reported methods.[2] The final DNA products were stored at −20 °C before use.

Single-molecule mechanical unfolding in optical tweezers

To construct coronazyme, we incubated 30 nM DNA construct prepared above with 3 nM 5 nm bare AuNP at −80 °C for 30 minutes, followed by thawing at room temperature (Figure S2).[1] The reaction mixture was diluted to 0.01 nM (DNA construct concentration) before experiments. To start mechanical unfolding, the DNA construct in the reaction mixture was bound to the anti-digoxigenin-coated beads by the digoxigenin/anti-digoxigenin linkage. Next, the DNA bound beads and the streptavidin-coated beads were separately injected into the top and bottom microchannels inside a three-channel microfluidic chamber. They were then flowed into the middle microchannel (Figure S3) to be trapped by two 1064 nm laser foci separately in a home-made optical tweezers instrument as described previously.[3] One laser beam was controlled through a steerable mirror to move one of the trapped beads away from another. This procedure allowed the free end of the DNA construct, modified with biotin, to attach to the streptavidin-coated bead via the biotin/streptavidin linkage. Once the two ends of the DNA construct were tethered to two different beads, it was stretched/relaxed by moving one bead away or towards from another. During this process, the force versus extension (F-X) traces were recorded, which were subsequently

analyzed by Labview software (National Instruments, TX). The loading rate during stretching/relaxing process was kept at ~5.5 pN/s (in the 10-30 pN force range).

Single-molecule fluorescent experiments by MT-HILO microscopy

The MT-HILO microscopy (Figure S4) was performed on an Olympus IX83 inverted microscope as described previously.[4] In short, two cubic magnets, with a 0.5 mm gap, were placed in an aluminum holder to apply magnetic force on superparamagnetic beads (diameter 2.8 μm, Life Technologies, Carlsbad, CA, USA). A 532 nm continuous laser beam (MGL-III-532, 300 mW, Dragon Laser, Changchun, Jilin, China) was adjusted via a Highly Inclined and Laminated Optical sheet (HILO) mirror to serve as the excitation source either with the epi-fluorescence and/or HILO modes.[5] To generate circularly polarized light (CPL), the polarized laser sequentially passed through a half waveplate and a quarter waveplate. By turning the quarter waveplate's angle to +45° or −45° with respect to the half waveplate, laser light was transformed into Right-Handed CPL (RHCP) or Left-Handed CPL (LHCP), respectively. The calibration of the tensile force applied to sample was accomplished according to previous publication.[4]

For single-molecule fluorescence experiment of coronazyme catalysis, we first covered 0.1% nitrocellulose on a Piranha solution cleaned glass coverslip. After the coverslip was dried, we heated it at 120 °C for 5 minutes and assembled it into a one-channel microfluidic chamber. Then, 50 μL 0.02 μg/μL anti-digoxigenin was injected and incubated for three hours. The coverslip surface was subsequently passivated by 50 μL 5 mg/mL BSA for 16 hrs. DNA samples (1 μL at 0.01 nM) were incubated with extra streptavidin-coated superparamagnetic beads (20 μL at 6-7 $\times 10^8$ beads/mL) for 30 minutes before injecting into the microfluidic chamber for another 30-minute incubation. Before recording movies of fluorescent signals of catalytic reactions, we rotated the magnets to confirm the rotation patterns of the magnetic bead, which are characteristic features for the single molecule DNA tether[4, 6] between the coverslip and the magnetic bead. The non-catalytic events occurring on the chamber surface were completely defocused due to MT-HILO design (see Section S4). The residue fluorescence background of each magnetic bead was photobleached for at least 30 minutes using the same 532 nm laser described above. When recording movies, the exposure time was 50 ms for each frame. Reaction buffer flow speed was 5 μL min^{-1} at 23 °C. Movies were treated in ThunderSTORM[7] and related steps & reaction rate calculations were described in literatures.[8]

Single-molecule fluorescent experiments of 5 nm bare AuNP

We employed an alternative method to detect the catalytic activity of 5 nm bare AuNPs. First, 100 µL of 20 pM solution of 5 nm bare AuNPs (nanoComposix, San Diego, CA, USA) was drop-cast onto a piranha solution-cleaned cover glass (VWR catalog#16004-096, 24 mm × 60 mm × thickness 0.13-0.16 mm) and allowed to incubate for 30 minutes at 23°C. Then, the AuNP-coated cover glass was rinsed with DI water for 2 minutes to remove any unbound AuNPs, followed by drying with nitrogen. By placing a layer of double-sided tape with a desired pattern on top of the AuNP-coated cover glass, a one-channel microfluidic chamber was prepared when the assembly was covered with another piece of cover glass (VWR 24 mm × 60 mm × thickness 0.13-0.16 mm) with two drilled holes. The chamber was further sealed with epoxy glue. The drilled holes allowed for the flow of the reaction buffer into and out of the microfluidic channel. Catalytic data were collected using a TIR fluorescence microscope,[8a] with a reaction buffer composed of 10 mM glucose, 0.5 µM AR, and 50% saturated oxygen in HEPES buffer (20 mM HEPES, 10 mM $MgCl_2$, 5 mM KCl, and 1 M NaCl at pH 7.5). During movie recording, the reaction buffer flowed at a rate of 5 µL min^{-1} at 23°C. The recorded movies were processed using ThunderSTORM[7-8] with additional details available in the literature.[8a]

UV melting and related kinetics calculation

We mixed 2 µM DNA glucose aptamer with and without 100 mM D-/L-glucose in a total volume of 600 µL using HEPES buffer (20 mM HEPES, 10 mM $MgCl_2$, 5 mM KCl, and 1 M NaCl at pH 7.5). The sample solutions were heated at 95 °C for 10 minutes, then rapidly cooled on ice. During UV melting experiments, the 260 nm absorbance of these samples was recorded using previous reports[9] and temperature ramping was 0.5 °C per minute (temperature range 15-85 °C). The folding (k_{on}) and unfolding (k_{off}) rates of DNA glucose aptamer with and without D-/L-glucose were calculated using literature reports (see SI section S5 for details).[10]

Materials

All chemicals, unless specified, were purchased from Thermo Fisher (www.thermofisher.com), Sigma Aldrich (www.sigmaaldrich.com) or VWR (www.vwr.com). DNA Oligos were obtained from Integrated DNA Technologies (www.idtdna.com) and purified by PAGE (sequence details see Table S1). L-glucose was purchased from Tokyo Chemical Industry (TCI, USA). The anti-digoxigenin/streptavidin coated polystyrene beads were bought from Spherotech (Lake Forest, IL, USA). The M-270 streptavidin-coated magnetic beads were acquired from Life Technologies (Carlsbad, CA, USA). The 5 nm bare gold nanoparticles (AuNP) were obtained from nanoComposix (San Diego, CA, USA). Nitrocellulose was bought from Cytiva Life Sciences (Marlborough, MA, USA). The DI water used to prepare the solutions and buffers was obtained from CANSHI® Ultrapure water system (Zhejiang Canshi, China).

Table S1. List of DNA oligonucleotides sequences

Name	Sequences (5'- to -3')	Function
Oligo I	GTC CGG ACC CTG TTTT CAG GGT CC	Terminal part of DNA corona
Oligo II	CTG TGA GGT AGT AGA AAA AAA AAA AAA AAA AAA AAC TTC AGT CTA GCG	polyA of DNA corona
Oligo III	GGA CCG CTA GAC TGA AGA AAA AAA AAA AAA AAA AAA AAC TAC TAC CTC	polyA of DNA corona
Oligo IV	CGG TAC GGT GTG AAA TAC CGC ACA GAT GCG ACG GTC GAT	Hairpin base of DNA corona
Oligo V	ACAG ATC GAC CGT GCC AGC AAG ACG TAG CCC AGC GCG TC	Hairpin base of DNA corona
Primer 1	CAG GGA CGC GCT GGG CTA CGT CTT GCT GGC	Link coronazyme and handles
Primer 2	CGC ATC TGT GCG GTA TTT CAC ACC GT	Link coronazyme and handles
Oligo I GA 1.0	GTC CGG **ACG ACC GTG TGT GTT GCT CTG TAA CAG TGT CCA TTG TCG** TCC	Glucose aptamer of DNA corona
Oligo I GA 2.0	GGC GCC **ACG ACC GTG TGT GTT GCT CTG TAA CAG TGT CCA TTG TCG** TGG	Glucose aptamer and GC-rich linker
Oligo II 2.0	CTG TGA GGT AGT AGA AAA AAA AAA AAA AAA AAA AAC GGG CCT TGG CGC	polyA, GC-rich linker and TT
Oligo III 2.0	CGC CGC GCC TTG GCC CGA AAA AAA AAA AAA AAA AAA AAC TAC TAC CTC	polyA, GC-rich linker and TT

S2. Syntheses of DNA samples and coronazymes

DNA sample synthesis

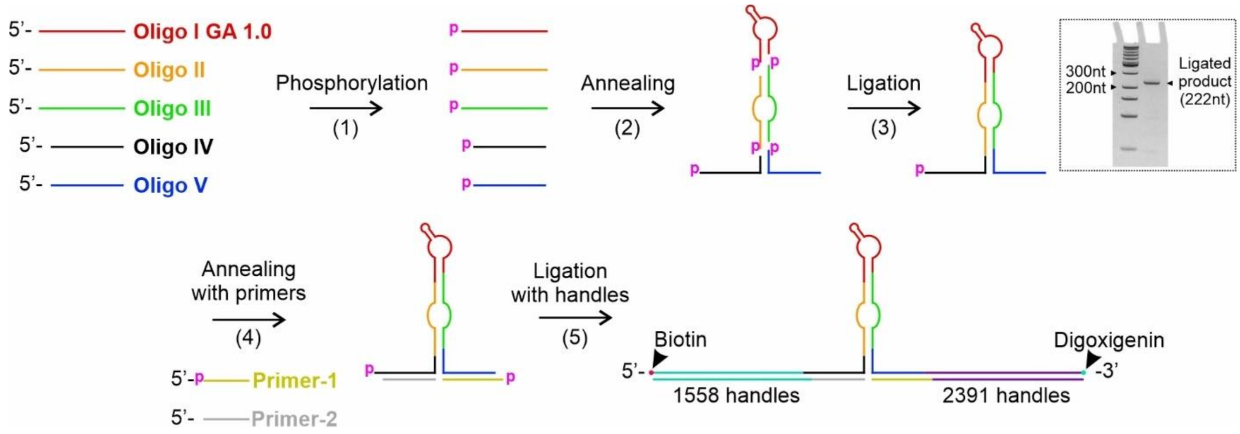

Figure S1. Preparation of corona DNA. (15% Native PAGE was used to confirm the ligated sample.)

To prepare DNA corona of the aptamer-coronazyme, we started with the phosphorylation of each of the following single-strand DNA (Oligos I GA 1.0, II, III, IV, and V, see Table S1). Next, Oligos II & III were mixed at 1:1 ratio, heated and gradually annealed from 95 to 20 °C at the rate of 0.5 °C/minute. Oligos IV & V followed the same annealing step. The Oligo I GA 1.0, the annealed Oligos II & III, and the annealed Oligos IV & V were then mixed at 1:1:1:1:1 ratio and ligated using T4 DNA ligase to form the corona DNA. To link corona DNA with DNA handles, the DNA corona was annealed with primer-2 and phosphorylated primer-1 (see Table S1), followed by one-pot ligation with 1558 bp handles[2b] and 2391 bp handles[2a] using T4 DNA ligase at 16 °C for 16 hrs. The product was stored at −20 °C before experiments.

The DNA corona of the Wild-Type (WT) coronazyme was made of Oligos I, II, III, IV and V (see Table S1).[1] The DNA corona of the G-C enriched aptamer-coronazyme was composed of Oligos I GA 2.0, II 2.0, III 2.0, IV and V (see Table S1). The syntheses of these two corona DNA pieces followed the same routes as described above.

Synthesis of coronazymes

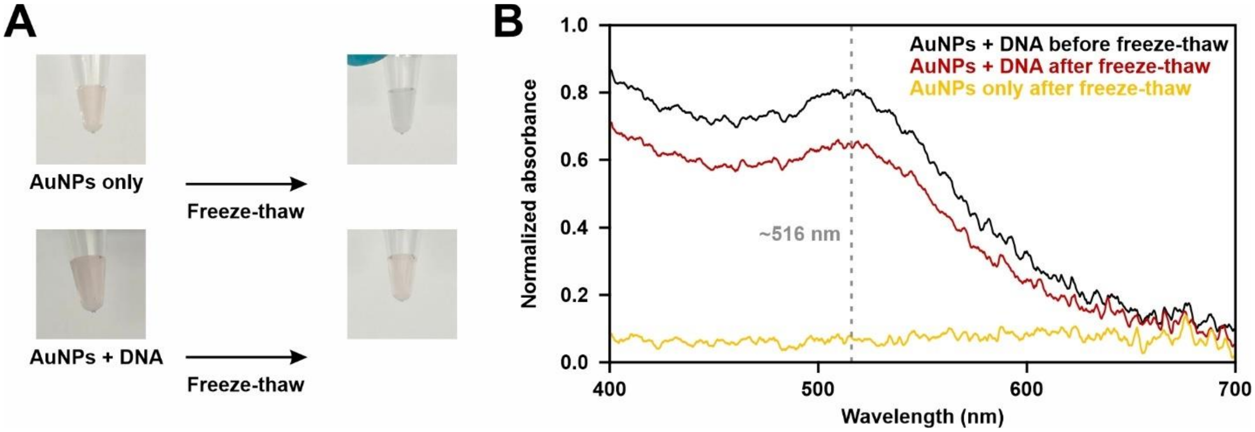

Figure S2. A) Images of color change before and after freeze-thaw of different samples. B) UV/Vis absorption spectra of AuNPs before (without DNA) and after freeze-thaw (DNA attached).

The aptamer-coronazymes were prepared by freeze-thaw procedures.[11] DNA corona samples and 5 nm bare AuNPs were mixed at 10:1 mole ratio and placed in −80 °C for 30 minutes. Then, the mixture was thawed at room temperature to facilitate the attachment of two poly(dA)$_{21}$ segments to the AuNP surface. Finally, the mixture solutions were stored at 4 °C before experiments.

The unchanged red color in the DNA-AuNP mixture solutions before and after freeze-thaw showed the success of the binding of two poly(dA)$_{21}$ segments to the AuNP (Figure S2A). The aborbance peak at ~516 nm in Figure S2B also supported this visual observation, demonstrating the success formation of coronazyme (AuNP coated with DNA corona). The grey color of the AuNP-only solution after the freeze-thaw procedures (Figure S2A) indicated the aggregation of AuNPs, resulting in the loss of 516 nm peak (Figure S2B).

S3. Microfluidic chamber for optical tweezers experiments

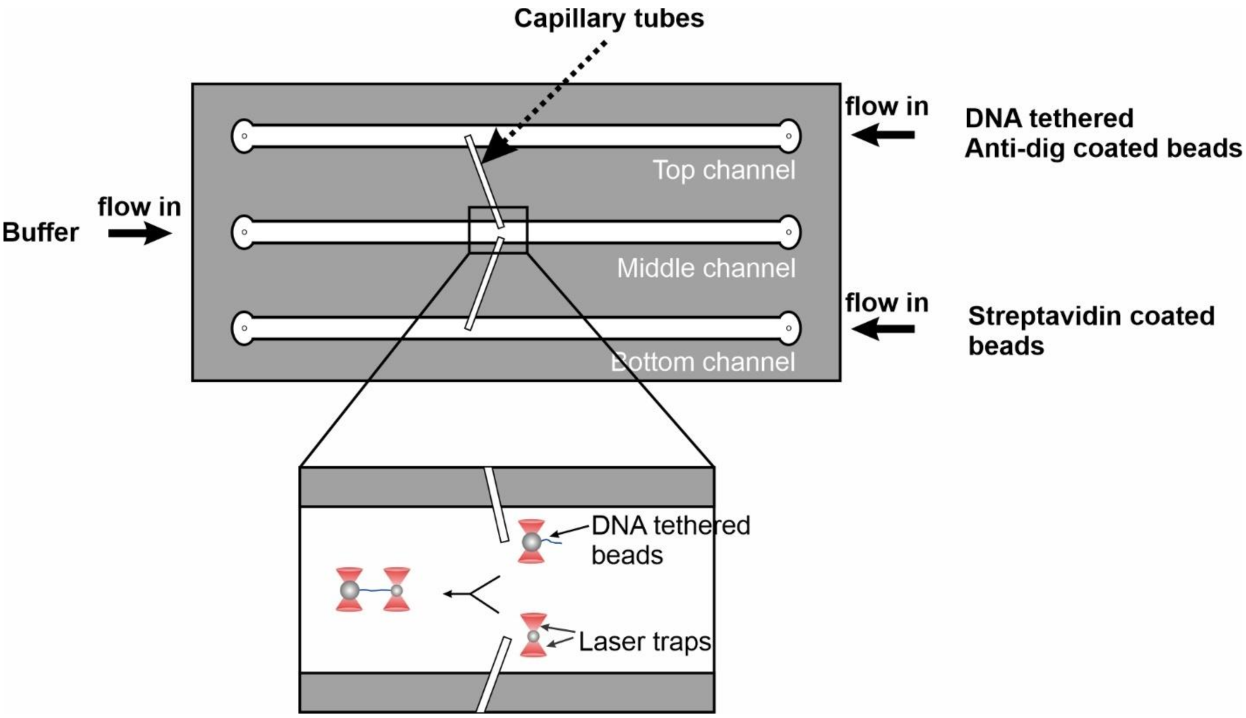

Figure S3. Schematic of a three-channel microfluidic chamber.

A three-channel microfluidic chamber (Figure S3) was used to perform single-molecular mechanical folding/unfolding experiments using optical tweezers. A paraffin film (Parafilm M, Bemis Laboratory Film, Bemis North America, WI, USA, thickness ~130 μm) with desired patterns was prepared by a laser cutter (VersaLaser®, Universal Systems, Inc., UT, USA). This film was placed between two pieces of cover glass (VWR catalog#16004-096, 24 mm × 60 mm × thickness 0.13-0.16 mm) with six holes punched in one cover glass by the same laser cutter. The whole setup was heated at 95 °C under a weight, making paraffin film tightly bond between the two coverslips. Between channels in chamber, capillary tubes (King Precision Glass, Inc, PO MC 7701, inner diameter 0.025 ± 0.010 mm) were placed to connect microfluidic channels.

For single-molecular mechanical folding/unfolding experiments, coronazyme samples linked with two DNA handles (see Section S2) were first mixed with anti-digoxigenin (dig) coated beads and incubated for 30 minutes at 23 °C to allow the attachment of the DNA construct to the beads. These beads were injected into the middle channel via the top channel (Figure S3). After

one of the beads was trapped by optical tweezers (see references[12] for detailed description of the optical tweezers instrument), the streptavidin-coated beads were introduced to the middle channel via the bottom channel (Figure S3). After one of the streptavidin coated beads was trapped by a second laser focus, the two trapped beads were brought closer to allow the tethering of a single coronazyme between the two beads, which was followed by force-ramping procedures for mechanical unfolding and folding experiments as described in literature.[13]

S4. Single-molecule fluorescent MT-HILO microscopy

The MT-HILO design significantly reduces background signals, because non-catalytic events occurring on the chamber surface are completely defocused.

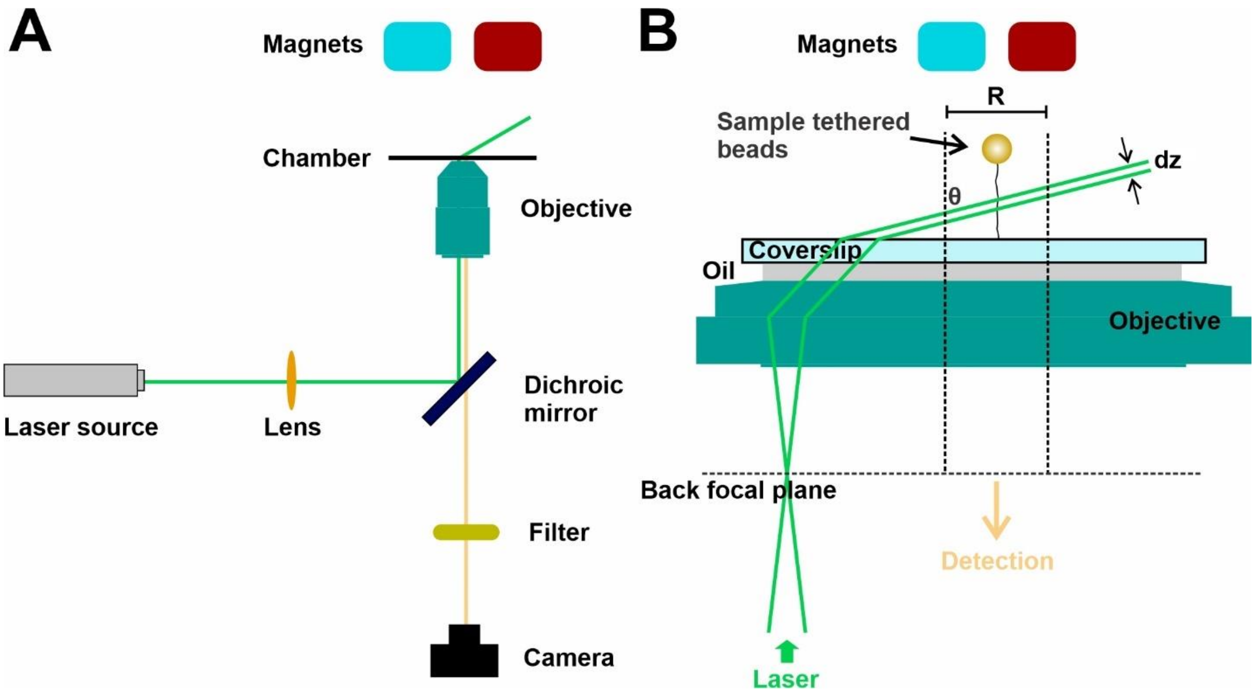

Figure S4. Setup of the MT-HILO microscope. A) Schematic illustration of the instrument layout. B) HILO excitation for a coronazyme. Here, R represents the diameter of the illuminated area, measured as 10 μm and θ shows the incidence angle at the coverslip, measured as ~70°. The dz reflects the laser illuminated thickness, which can be calculated by $dz = \frac{R}{\tan\theta}$ and the value of dz is ~3.6 μm.

S5. The folding (k_{on}) and unfolding (k_{off}) rate constants calculations

Other than determining melting temperature (T_m), it is also possible to extract kinetic information, such as the folding (k_{on}) and unfolding (k_{off}) rate constants, in UV melting experiments.[10] To achieve this, the absorbance vs. temperature data obtained from UV melting experiments were transformed into fraction folded (θ_T) *vs*. temperature relationship:[10]

$$\theta_T = (L0_T - A_T)/(L0_T - L1_T) \qquad \text{(Equation S1)},$$

where A_T represents the absorbance at temperature T. $L0_T$ and $L1_T$ are baseline values of unfolded and folded species at temperature T, respectively.

During the cooling/heating cycles of DNA samples with and without a ligand such as glucose, the kinetic constants of structural folding (k_{on}) and unfolding (k_{off}) at each temperature were obtained following equation S2[10]:

$$d(\theta)/dt = k_{on}\theta - k_{off}(1 - \theta) \qquad \text{(Equation S2)},$$

Where θ is obtained by Equation S1 and d(θ)/dt represents the rate of θ. To solve the equation S2 and find two unknowns (k_{on} and k_{off}), we used $\theta_{heating}$ and $d(\theta_{heating})/dt$ values from heating process as well sa $\theta_{cooling}$, and $d(\theta_{cooling})/dt$ values from cooling process, separately, which give equations S3 and S4,[10]

$$d(\theta_{heating})/dt = k_{on}\theta_{heating} - k_{off}(1 - \theta_{heating}) \qquad \text{(Equation S3)},$$

$$d(\theta_{cooling})/dt = k_{on}\theta_{cooling} - k_{off}(1 - \theta_{cooling}) \qquad \text{(Equation S4)}.$$

Finally, the numerical values for k_{on} and k_{off} at each temperature point were obtained.

S6. DNA corona structure of aptamer-coronazyme

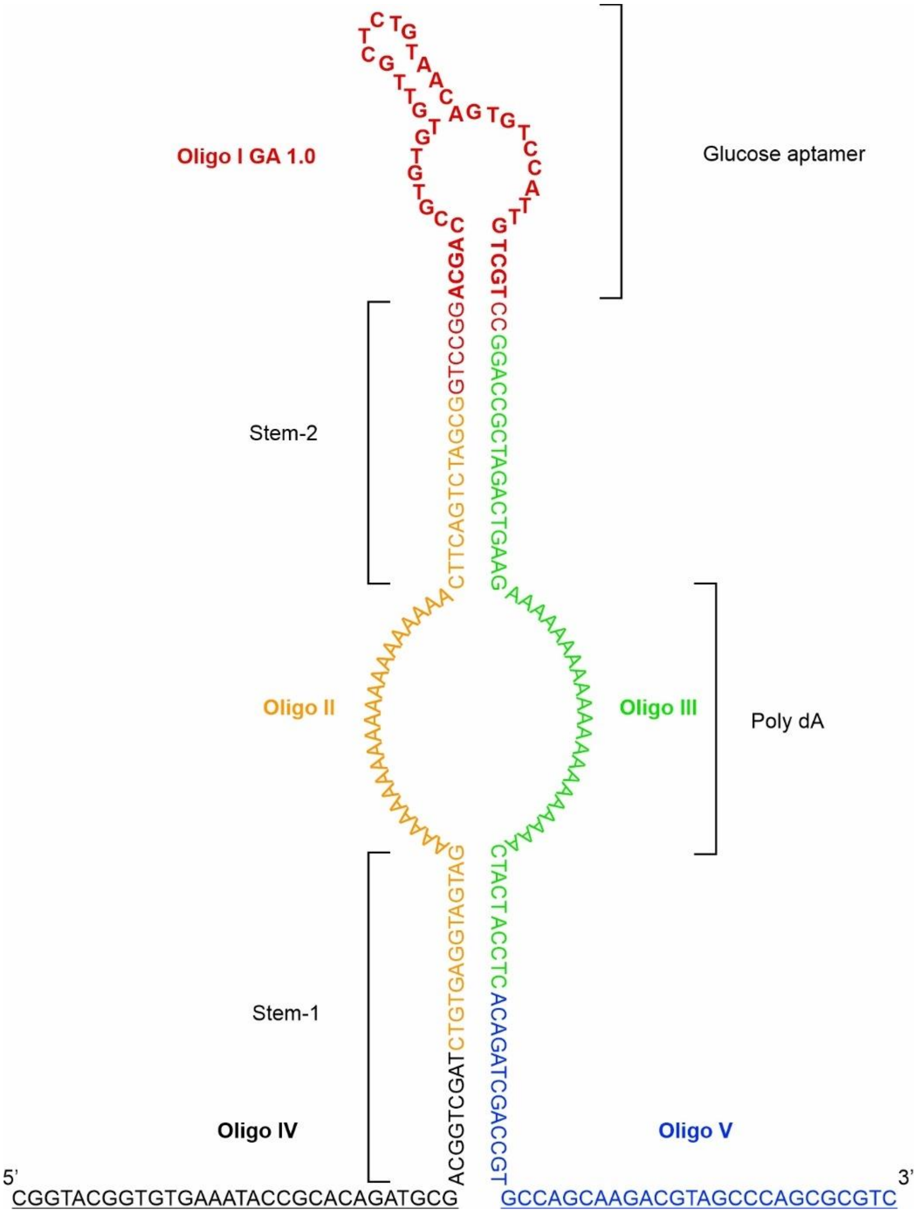

Figure S5. The complete structure of DNA corona in aptamer-coronazyme. The DNA corona is made of 5 pieces of single-stranded DNA represented by different colors. The underlined parts of Oligos IV&V are used to anneal with primers 1&2 to ligate with 1558 bp or 2391 bp handles (see section S2), respectively. The two poly(dA)$_{21}$ segments of DNA corona form a clamp-like structure with a diameter of approximately 7 nm, which is well-suited for binding to a 5 nm AuNP.

S7. Mechanical unfolding results correlated with glucose binding

To confirm the binding of glucose to the glucose aptamer (Figure 2B), we unfolded the DNA corona of aptamer-coronazymes in the presence of D-/L-glucose using optical tweezers. (Figure S6).

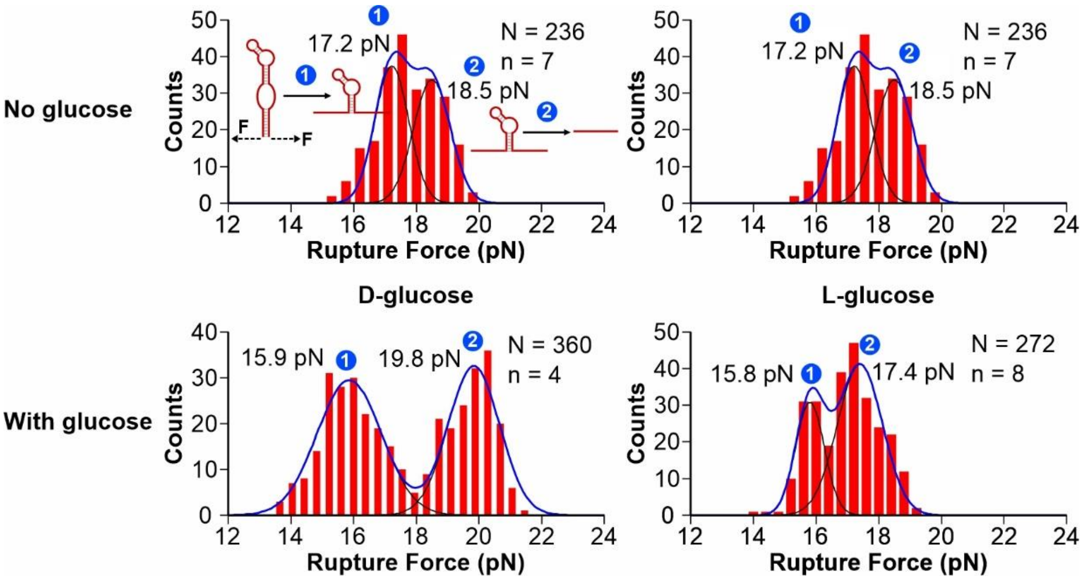

Figure S6. Unfolding force histograms of corona DNA from aptamer-coronazymes with and without 100 mM D-/L-glucose.

The rupture force of Step I was 17.2 pN, while Step II, containing the glucose aptamer, exhibited a rupture force of 18.5 pN in the absence of glucose. In the presence of 100 mM D-/L-glucose, the rupture force of Part I decreased (15.9 pN with D-glucose, 15.8 pN with L-glucose), indicating that high glucose concentrations weakened the DNA structure, consistent with previous reports.[14]

For Step II, the rupture force increased from 18.5 pN (without glucose) to 19.8 pN (100 mM D-glucose), demonstrating binding-induced structural changes. In contrast, exposure to 100 mM L-glucose led to a decrease in rupture force to 17.4 pN, suggesting weaker glucose-binding-induced structural changes alongside significant glucose-induced DNA weakening. The melting temperatures follow these trends (T_m of 62.0 °C (D-glucose), 58.0 °C (L-glucose), and 60.1 °C (without glucose)).

S8. Expected ΔL calculations

The expected change-in-contour-length (ΔL) is calculated based on the following equation:

$$\Delta L = (N \times L_{\mathrm{nt}}) - x \qquad \text{(Equation S5)},$$

where N is the number of nucleotides in the DNA sample, L_{nt} is the average contour-length per nucleotide (0.40 - 0.48 nm)[15], and x is the end-to-end distance of folded DNA secondary structures (x is 2 nm for double-strand DNA[16]).

For the DNA corona of aptamer-coronazymes, the expected ΔL of the stem-1 and poly dA, annotated as Part I of Figure 2A (also see Figure S5), is ~ 35-42 nm. Our experimental data was 43.1 nm, close to the expected value. The expected ΔL of the stem-2 and glucose aptamer, annotated as Part II of Figure 2A (also see Figure S5), is ~29-32 nm, close to our experimental result of 28.4 nm.

S9. DNA corona structure of G-C enriched aptamer-coronazyme

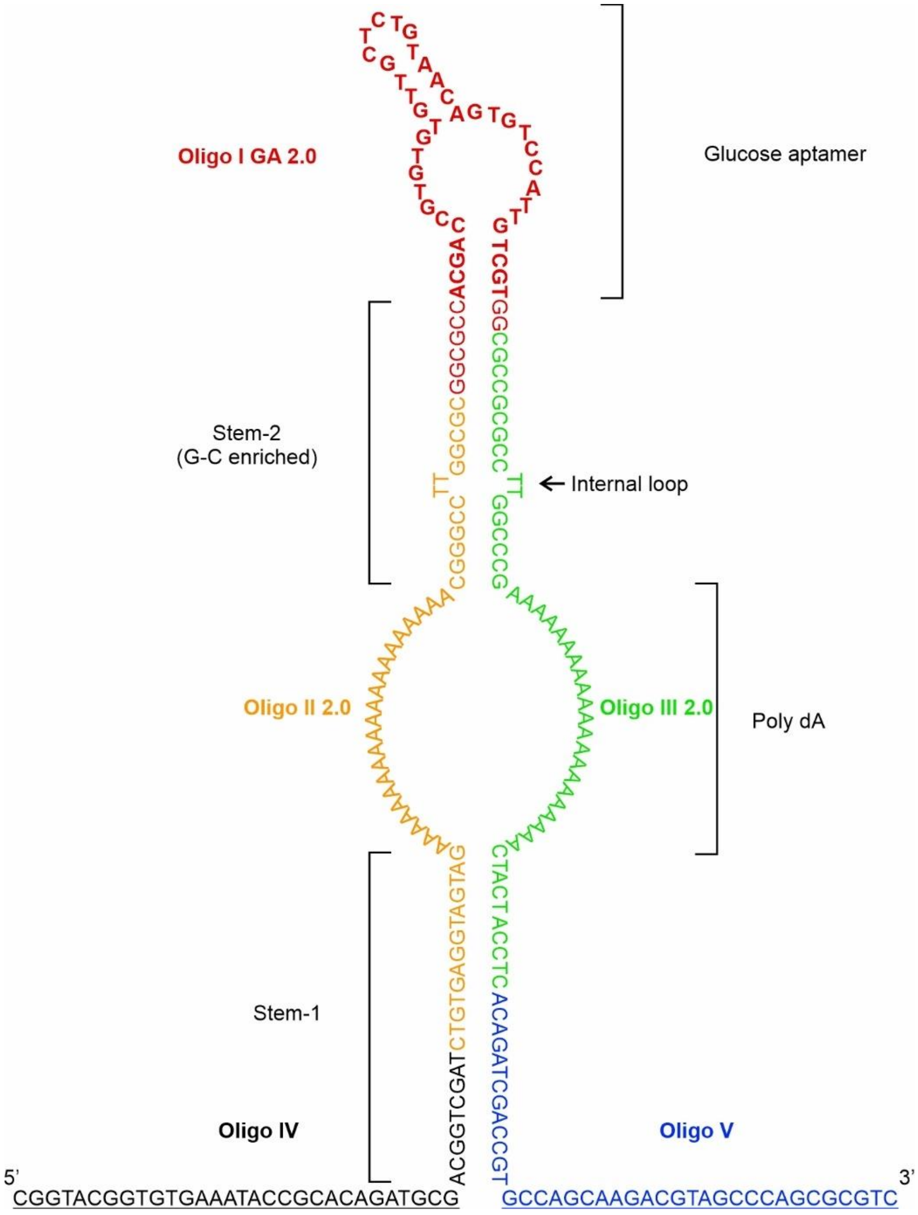

Figure S7. The complete structure of DNA corona in a G-C enriched aptamer-coronazyme. The DNA corona is made of five single-stranded DNA segments represented by different colors. The underlined parts of Oligos IV&V are used to anneal with primers 1&2 to ligate with 1558 bp and 2391 bp handles (see section S2), respectively.

S10. Enzymatic stability test

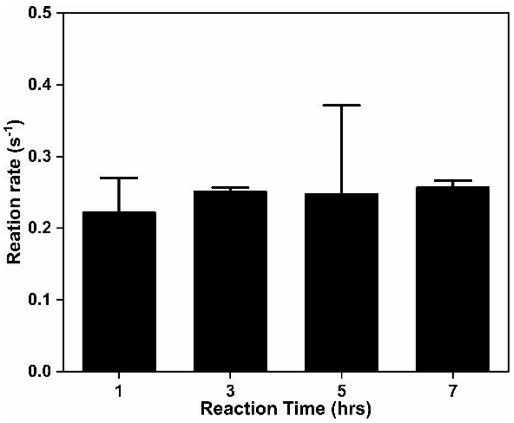

Figure S8. Enzymatic stability of the aptamer-coronazyme with 10 mM D-glucose under RHCP. The average reaction rates for aptamer-coronazymes were determined after 1-hour, 3-hour, 5-hour and 7-hour reaction time. The error bars depicted the standard deviations.

S11. Spin polarization calculation

The spin polarization is determined using Mott polarimetry measurements:

$$p = (I_+ - I_-)/(I_+ + I_-) \qquad \text{(Equation S6)},$$

where p is the spin polarization, and I_+ and I_- represent the signal intensities with spin orientations parallel and antiparallel to the electron velocity, respectively.[17]

Because catalytic reactivity depends on photoelectrons, a reactivity-based equation (Equation S7) can be used in a similar form to measure spin polarization:

$$p = (\nu_{\mathrm{RHCP}} - \nu_{\mathrm{LHCP}})/(\nu_{\mathrm{RHCP}} + \nu_{\mathrm{LHCP}}) \qquad \text{(Equation S7)},$$

where p was the spin polarization, and ν_{RHCP} and ν_{LHCP} are the reaction rates of the catalyst (e.g., with glucose) under RHCP and LHCP illumination, respectively.

Similar variants of these calculation formulas have been reported in the literature.[18]

S12. Electron spin change during glucose catalysis

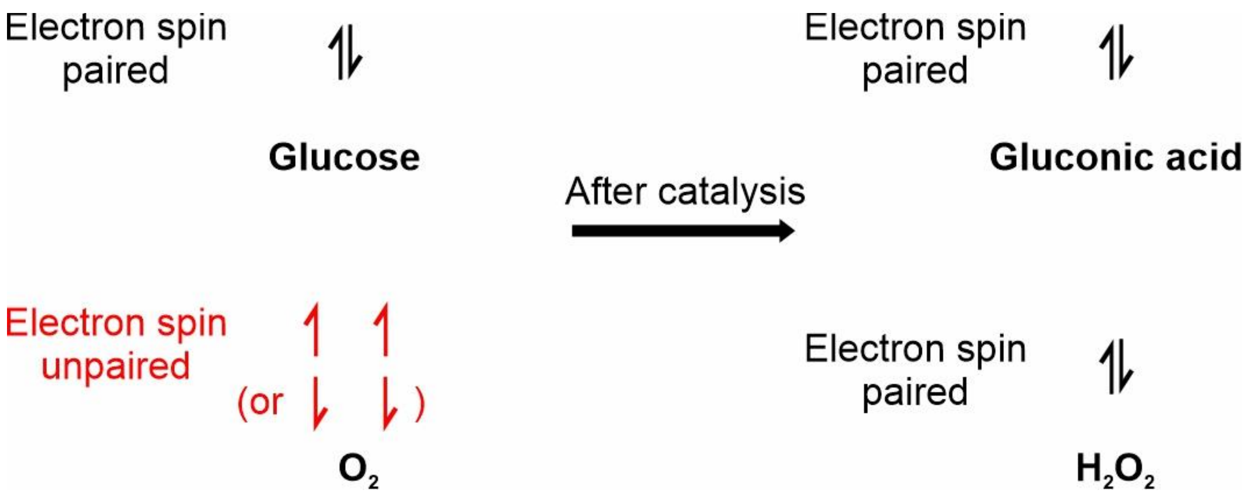

Figure S9. The electron spin states before and after the glucose oxidation reaction catalyzed by the coronazyme.

S13. References

[1] P. Pokhrel, K. Ren, H. Shen, H. Mao, *Langmuir* **2022**, *38*, 13569.

[2] a) S. Pandey, Y. Xiang, D. Friedrich, Y. Leng, H. Mao, *The Journal of Physical Chemistry Letters* **2021**, *12*, 11316; b) S. Pandey, S. Mandal, M. B. Danielsen, A. Brown, C. Hu, N. J. Christensen, A. V. Kulakova, S. Song, T. Brown, K. J. Jensen, J. Wengel, C. Lou, H. Mao, *Nature Communications* **2022**, *13*, 76.

[3] D. Koirala, S. Dhakal, B. Ashbridge, Y. Sannohe, R. Rodriguez, H. Sugiyama, S. Balasubramanian, H. Mao, *Nat. Chem.* **2011**, *3*, 782.

[4] L. Zuo, J. Ji, P. Pokhrel, B. Pokhrel, K. Ren, H. Mao, H. Shen, *ChemRxiv* **2023**.

[5] a) J. Tang, K. Y. Han, *Optica* **2018**, *5*, 1063; b) M. Tokunaga, N. Imamoto, K. Sakata-Sogawa, *Nature Methods* **2008**, *5*, 159.

[6] C. Liu, K. Kubo, E. Wang, K.-S. Han, F. Yang, G. Chen, F. A. Escobedo, G. W. Coates, P. Chen, *Science* **2017**, *358*, 352.

[7] a) B. Bintu, L. J. Mateo, J.-H. Su, N. A. Sinnott-Armstrong, M. Parker, S. Kinrot, K. Yamaya, A. N. Boettiger, X. Zhuang, *Science* **2018**, *362*, eaau1783; b) M. Ovesný, P. Křížek, J. Borkovec, Z. Švindrych, G. M. Hagen, *Bioinformatics* **2014**, *30*, 2389.

[8] a) L. Zuo, K. Ren, X. Guo, P. Pokhrel, B. Pokhrel, M. A. Hossain, Z.-X. Chen, H. Mao, H. Shen, *Journal of the American Chemical Society* **2023**, *145*, 5750; b) W. Xu, J. S. Kong, P. Chen, *The Journal of Physical Chemistry C* **2009**, *113*, 2393.

[9] A. V. Tataurov, Y. You, R. Owczarzy, *Biophysical Chemistry* **2008**, *133*, 66.

[10] J. L. Mergny, L. Lacroix, *Oligonucleotides* **2003**, *13*, 515.

[11] M. Hu, C. Yuan, T. Tian, X. Wang, J. Sun, E. Xiong, X. Zhou, *Journal of the American Chemical Society* **2020**, *142*, 7506.

[12] a) Z. Yu, J. D. Schonhoft, S. Dhakal, R. Bajracharya, R. Hegde, S. Basu, H. Mao, *J. Am. Chem. Soc.* **2009**, *131*, 1876; b) J. D. Schonhoft, R. Bajracharya, S. Dhakal, Z. Yu, H. Mao, S. Basu, *Nucleic Acids Res.* **2009**, *37*, 3310.

[13] Z. Yu, H. Mao, *Chem. Rec.* **2013**, *13*, 102.

[14] M. Lorenzi, D. F. Montisano, S. Toledo, A. Barrieux, *The Journal of clinical investigation* **1986**, *77*, 322.

[15] a) Z. Yu, V. Gaerig, Y. Cui, H. Kang, V. Gokhale, Y. Zhao, L. H. Hurley, H. Mao, *J. Am. Chem. Soc.* **2012**, *134*, 5157; b) M. T. Woodside, W. M. Behnke-Parks, K. Larizadeh, K. Travers, D. Herschlag, S. M. Block, *Proc. Natl. Acad. Sci. U S A* **2006**, *103*, 6190; c) T. A. Laurence, X. Kong, M. Jager, S. Weiss, *Proc. Nat. Acad. Sci. USA* **2005**, *102*, 17348; d) J. B. Mills, E. Vacano, P. J. Hagerman, *J. Mol. Biol.* **1999**, *285*, 245.

[16] R. R. Sinden, *DNA structure and function*, Academic Press, San Diego, CA, **1995**.

[17] K. Chae, N. A. R. C. Mohamad, J. Kim, D.-I. Won, Z. Lin, J. Kim, D. H. Kim, *Chemical Society Reviews* **2024**.

[18] N. Preeyanka, Q. Zhu, T. K. Das, R. Naaman, *ChemPhysChem* **2024**, *25*, e202400033.
